# Examining the mediating function of SIRI in the association between LC9 and chronic obstructive pulmonary disease

**DOI:** 10.3389/fmed.2025.1606864

**Published:** 2025-07-09

**Authors:** Ziming Wang, Xinyun Xie, Hongyang Gong

**Affiliations:** ^1^Department of Thoracic Surgery, Shanghai Pulmonary Hospital, School of Medicine, Tongji University, Shanghai, China; ^2^Department of Respiratory and Oncology, Chengdu Sixth People’s Hospital, Chengdu, Sichuan, China; ^3^Department of Physiology, College of Medicine, Chosun University, Gwangju, Republic of Korea

**Keywords:** Life’s Crucial 9, chronic obstructive pulmonary disease, systemic inflammation response index, NHANES, mediation analysis

## Abstract

**Background:**

Chronic obstructive pulmonary disease (COPD) is a major contributor to early mortality, high overall mortality rates, and significant healthcare costs. Based on the Life’s Essential 8 (LE8), Life’s Crucial 9 (LC9) is a new cardiovascular health evaluation instrument that also includes a mental health examination, while the systemic inflammation response index (SIRI) is an emerging biomarker of systemic inflammation. The purpose of this study is to look at the link between LC9 and COPD and how SIRI may play a mediating function in it.

**Methods:**

Data from 25,721 U.S. participants in the National Health and Nutrition Examination Survey (NHANES) from 2005 to 2018 were used in a cross-sectional study. To investigate the relationship between COPD and LC9, multivariable logistic regression, restricted cubic splines (RCS), and subgroup analysis were used. Furthermore, a mediation study was conducted to evaluate SIRI’s possible involvement in the connection between LC9 and COPD.

**Results:**

It consisted of 25,721 persons in all, 1,257 of whom had COPD. When multiple covariates were taken into account, logistic regression analysis showed that a 28% decrease in the prevalence of COPD was linked to every 10-point increase in LC9 (OR = 0.72, 95% CI: 0.67, 0.77), while a 1.17-fold increase in the prevalence of COPD was linked to each unit increase in SIRI (OR = 1.17, 95% CI: 1.10, 1.25). When LC9 and SIRI were divided into tertiles, similarities were seen (P for trend <0.001). A linear negative correlation between LC9 and the prevalence of COPD was shown using RCS analysis. Also, mediation analysis indicated that SIRI mediated 1.64% of the link between LC9 and COPD (*p* < 0.001).

**Conclusion:**

As shown by this study, LC9 and COPD display a substantial negative connection, which is largely mediated by SIRI. These results demand more investigation because they show a possible connection between cardiovascular health and COPD and offer fresh perspectives on the role systemic inflammation plays in this relationship.

## Introduction

Chronic obstructive pulmonary disease (COPD) is a chronic respiratory disorder characterized by persistent inflammation, airway remodeling, and immune activation in the lung parenchyma. Globally, COPD accounts for over 3 million deaths annually, making it the third leading cause of mortality. It is estimated to affect more than 10% of the population, with a steadily increasing prevalence (1). COPD is frequently accompanied by comorbid conditions, including depression, osteoporosis, and cardiovascular diseases, which collectively reduce overall health and life quality of those who are impacted.

Investigation has revealed that cardiovascular comorbidities and COPD have a significant association as they share common risk factors, pathophysiological mechanisms, symptoms, and clinical manifestations (2). Life’s Crucial 9 (LC9), a cardiovascular health score recently introduced by the American Heart Association, builds upon Life’s Essential 8 (LE8) by incorporating mental health as an additional component. LC9 consists of nine key metrics: diet, physical activity, smoking cessation (nicotine exposure), sleep health, body mass index (BMI), blood lipids, blood glucose, blood pressure, and mental well-being. While LC9 has been linked to cardiovascular events, recent studies have also reported associations between LC9 and conditions such as overactive bladder (OAB) (3) and infertility among U.S. women (4). Given the high prevalence of cardiovascular co-morbidities in COPD and the multifactorial nature of COPD risk factors as well as the common physiopathological mechanisms of inflammation in cardiorespiratory diseases, whereas previous studies have only been limited to exploring the analysis of the association of LC9 with COPD and the potential mediating role of lean body mass (LBM) in the association of LC9 with COPD (5, 6), while neglecting the importance of inflammation. It is therefore crucial to explore the association of LC9 with COPD and the underlying mechanisms of its inflammation.

A new inflammatory biomarker based on the numbers of monocytes, neutrophils, and lymphocytes is the systemic inflammation response index (SIRI). Chronic airway inflammation is a hallmark of COPD, often accompanied by systemic inflammation, which has been associated with adverse outcomes such as recurrent acute exacerbations of COPD (AECOPD) (7). Emerging evidence suggests that SIRI is associated with all-cause and cardiovascular mortality in chronic kidney disease (CKD) patients (8), increased stroke prevalence in individuals with asthma (9), and higher mortality in adult asthma patients (10). A recent study also identified SIRI as an independent risk factor for COPD (11). This study postulates that SIRI mediates the relationship between LC9 and COPD, considering these findings. Utilizing data from the 2005–2018 NHANES cycles, this study aims to investigate the interrelationships among SIRI, LC9, and COPD.

## Methods

### Study participants

A continuous, nationally representative study, the National Health and Nutrition Examination Study (NHANES), evaluates the health and nutritional status of Americans. NHANES provided the data for this cross-sectional investigation. The National Center for Health Statistics’ (NCHS) Ethics Review Board approved all study protocols before data collection, and each participant gave written informed consent. This secondary analysis adheres to the STROBE guidelines for cross-sectional studies (12) and does not require additional Institutional Review Board (IRB) approval.

Further methodological details can be found at https://www.cdc.gov/nchs/nhanes/index.htm.

**Figure 1:**
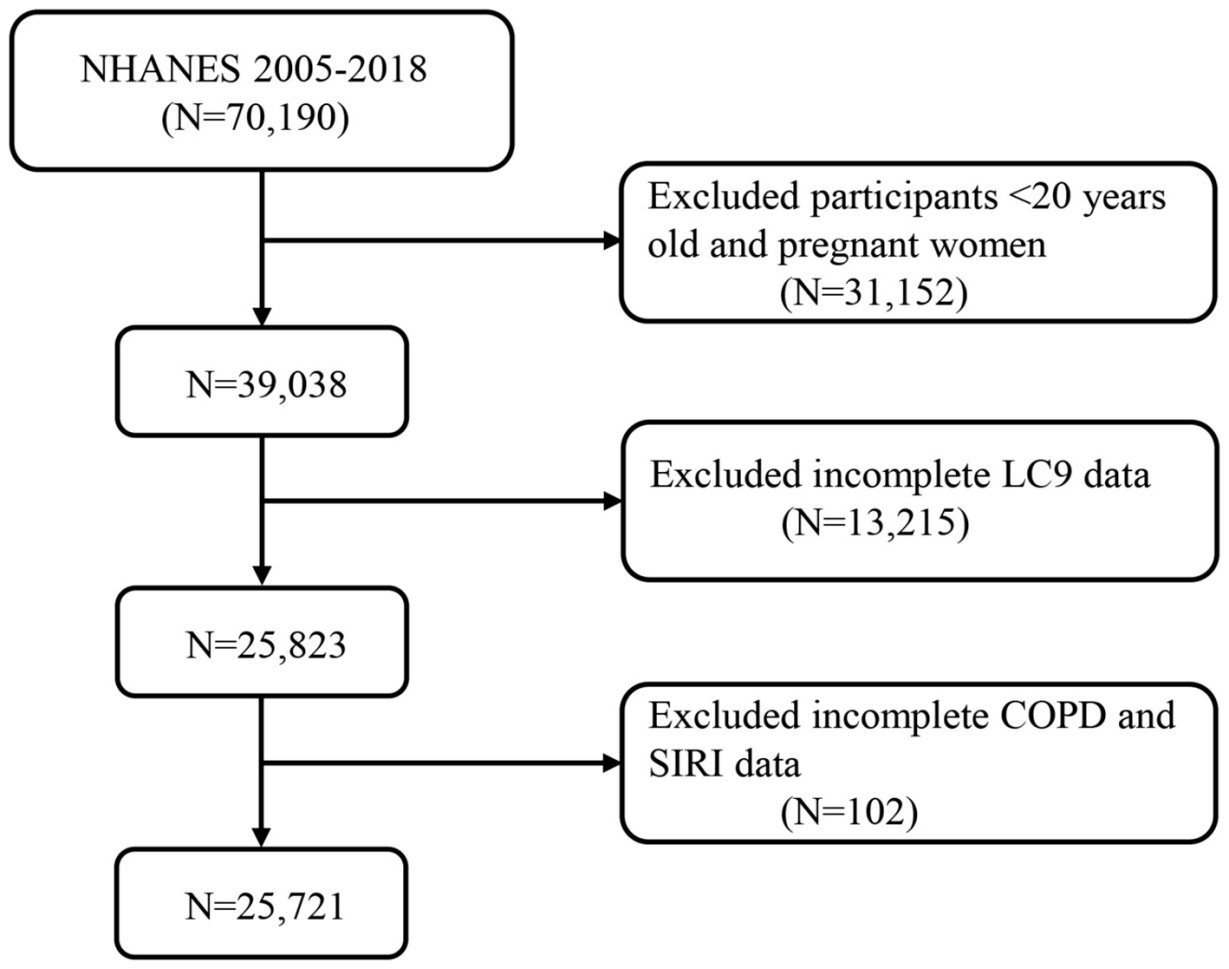
A flow diagram of eligible participant selection in the National Health and Nutrition Examination Survey. LC9, Life’s Crucial 9; SIRI, systemic inflammation response index; COPD, chronic obstructive pulmonary disease.

Data from NHANES, which was nationally representative, was adopted in this cross-sectional investigation. A total of 70,190 participants were initially identified from seven NHANES cycles (2005–2018). Participants were excluded if they were under 20 years of age or pregnant (*n* = 31,152), had missing LC9 data (*n* = 13,215), or had incomplete SIRI or COPD data (*n* = 102). Ultimately, 25,721 participants were included in the final analysis (Figure 1).

### Definition of SIRI

Using a Coulter^®^ DxH 800 automated hematology analyzer, the complete blood count (CBC), which includes neutrophil, lymphocyte, and monocyte counts, was determined. The formula used to compute the systemic inflammatory response index (SIRI) was SIRI = neutrophil count × monocyte count/lymphocyte count (11).

### Diagnosis of COPD

The following criteria were used in this investigation to diagnose COPD (13): 1. Forced expiratory volume in one second (FEV1)/forced vital capacity (FVC) < 0.7 after a bronchodilator; 2. A physician or other healthcare professional diagnosed the participant with COPD; 3. A physician or other healthcare professional diagnosed the participant with emphysema or chronic bronchitis; 4. Participants had to be at least 40 years old, have a history of smoking, suffer from chronic bronchitis, and be using leukotriene modulators, mast cell stabilizers, selective phosphodiesterase-4 inhibitors, or inhaled corticosteroids.

### Definition of Life’s Crucial 9

The nine elements of LC9 include five health variables (weight management, blood glucose management, blood pressure management, cholesterol control, and mental health) and four health habits (physical activity, quitting smoking, healthy eating, and sleep health). Supplementary Table S1 contains comprehensive instructions on how to compute LC9 scores from NHANES data. In short, each of the nine LC9 components is assigned a number between 0 and 100, and the average of these nine ratings is used to get the total LC9 score. The Healthy Eating Index-2015 (HEI-2015) was used to assess dietary health (14), with its components and scoring criteria detailed in Supplementary Table S2. Sleep health, smoking status, physical activity, and mental health were derived from standardized questionnaires, while BMI, blood pressure, blood glucose, and cholesterol levels were measured by trained professionals using NHANES data.^1^

### Covariables

Adjustments were conducted for age, sex, race/ethnicity, marital status, education level, poverty income ratio (PIR), smoking status, alcohol intake, hypertension, diabetes, and hyperlipidemia to increase the findings’ robustness. Supplementary Table S3 offers thorough explanations of these variables.

### Statistical analysis

Statistical analyses were conducted using R software (version 4.3.1). To ensure national representativeness, all analyses were weighted according to NHANES sampling procedures. Continuous variables are presented as means ± standard deviation (SD), while categorical variables are presented as frequencies (percentages). Weighted *t*-tests and weighted chi-square tests were used to compare continuous and categorical variables, respectively. Multivariable logistic regression models were employed to assess the associations between LC9 and COPD, as well as between SIRI and COPD. We built three models: First Model: Unmodified; Age, sex, education, marital status, PIR, and race/ethnicity were all considered in Model 2; Model 3: Enhanced for diabetes, hyperlipidemia, and hypertension. LC9, SIRI, and COPD were examined for any nonlinear connections using smooth curve fitting. Risk stratification for the relationship between LC9 and COPD was evaluated across several categories using subgroup analysis. To assess SIRI’s direct, indirect, and overall impacts on the association between LC9 and COPD, a mediation analysis was conducted. Mediation proportion = Indirect impact/(Indirect effect + Direct effect) × 100% was the formula used to determine the percentage of mediation. Using R’s “mediation” package, the mediation effect was calculated (11). *p*-values < 0.05 were regarded as statistically significant.

## Results

### Baseline characteristics

25,721 people who were 20 years of age or older participated in this study, representing an estimated 121,031,208 adults in the United States. The prevalence of COPD was 4.8%, corresponding to approximately 5,852,634 individuals. Age, race, income, education, hypertension, diabetes, and hyperlipidemia all showed significant differences between those with and without COPD (*p* < 0.05). Those with COPD were more likely to be married and male than those without the disease. Additionally, LC9 scores were lower in the COPD group than in the non-COPD group, whereas SIRI levels were higher in the COPD group. Table 1 lists the specific characteristics. The baseline characteristics of the unweighted population are shown in Supplementary Table S4.

**Table 1:** Baseline characteristics of all participants were stratified by COPD, weighted.

Characteristic	Overall, *N* = 121,031,208 (100%)	Non-COPD, *N* = 115,178,574 (95.2%)	COPD, *N* = 5,852,634 (4.8%)	*p* value
No. of participants in the sample	25,721	24,464	1,257	–
Age (%)				**<0.001**
20–40	42,302,663 (35%)	41,986,502 (36%)	316,161 (5.4%)	
41–60	45,612,559 (38%)	43,251,290 (38%)	2,361,269 (40%)	
>60	33,115,986 (27%)	29,940,782 (26%)	3,175,204 (54%)	
Sex (%)				0.322
Female	62,272,296 (51%)	59,387,363 (52%)	2,884,933 (49%)	
Male	58,758,912 (49%)	55,791,212 (48%)	2,967,700 (51%)	
Race (%)				**<0.001**
Non-Hispanic White	85,883,927 (71%)	80,974,636 (70%)	4,909,291 (84%)	
Non-Hispanic Black	12,330,526 (10%)	11,972,712 (10%)	357,813 (6.1%)	
Other	13,916,977 (11%)	13,430,957 (12%)	486,020 (8.3%)	
Mexican American	8,899,778 (7.4%)	8,800,269 (7.6%)	99,509 (1.7%)	
Married/live with partner (%)				0.539
No	43,131,433 (36%)	41,108,204 (36%)	2,023,228 (35%)	
Yes	77,867,344 (64%)	74,037,939 (64%)	3,829,405 (65%)	
Education level (%)				**0.001**
Below high school	16,873,479 (14%)	15,785,819 (14%)	1,087,661 (19%)	
High school or above	104,120,741 (86%)	99,355,768 (86%)	4,764,973 (81%)	
PIR (%)				**0.004**
Poor	22,010,138 (19%)	20,722,155 (19%)	1,287,983 (24%)	
Not poor	91,711,501 (81%)	87,544,248 (81%)	4,167,253 (76%)	
Hypertension (%)				**<0.001**
No	73,807,556 (61%)	71,319,907 (62%)	2,487,649 (43%)	
Yes	47,223,652 (39%)	43,858,667 (38%)	3,364,985 (57%)	
Diabetes (%)				**<0.001**
No	105,630,984 (87%)	101,095,382 (88%)	4,535,602 (77%)	
Yes	15,400,224 (13%)	14,083,192 (12%)	1,317,031 (23%)	
Hyperlipidemia (%)				**<0.001**
No	34,266,129 (28%)	33,277,105 (29%)	989,024 (17%)	
Yes	86,765,079 (72%)	81,901,469 (71%)	4,863,610 (83%)	
Mean LC9 score (mean (SD))	70.43 (13.61)	70.81 (13.50)	62.96 (13.76)	**<0.001**
LC9, Tertile (%)				**<0.001**
T1	39,243,624 (32%)	36,186,112 (31%)	3,057,512 (52%)	
T2	40,915,899 (34%)	39,144,629 (34%)	1,771,270 (30%)	
T3	40,871,685 (34%)	39,847,833 (35%)	1,023,851 (17%)	
Mean psychological health score (mean (SD))	89.23 (23.32)	89.50 (23.00)	83.99 (28.42)	**<0.001**
Mean HEI-2015 diet score (mean (SD))	39.52 (31.45)	39.55 (31.49)	38.94 (30.52)	0.691
Mean physical activity score (mean (SD))	71.70 (41.07)	72.22 (40.75)	61.36 (45.60)	**<0.001**
Mean tobacco exposure score (mean (SD))	71.33 (38.60)	72.39 (38.27)	50.45 (39.24)	**<0.001**
Mean sleep health score (mean (SD))	83.45 (24.21)	83.66 (24.00)	79.23 (27.63)	**<0.001**
Mean body mass index score (mean (SD))	60.54 (33.51)	60.66 (33.50)	58.21 (33.66)	0.066
Mean blood lipid score (mean (SD))	63.91 (30.32)	64.19 (30.33)	58.34 (29.65)	**<0.001**
Mean blood glucose score (mean (SD))	85.58 (24.21)	86.06 (23.94)	76.22 (27.55)	**<0.001**
Mean blood pressure score (mean (SD))	68.59 (31.13)	69.03 (31.10)	59.93 (30.57)	**<0.001**
SIRI (mean (SD))	1.26 (0.86)	1.24 (0.84)	1.55 (1.16)	**<0.001**
SIRI, Tertile (%)				**<0.001**
T1	40,320,395 (33%)	39,001,724 (34%)	1,318,671 (23%)	
T2	40,397,142 (33%)	38,455,361 (33%)	1,941,781 (33%)	
T3	40,313,671 (33%)	37,721,490 (33%)	2,592,181 (44%)	

Mean (SD) for continuous variables: the *p* value was calculated by the weighted Students *T*-test.

Percentages (weighted *N*, %) for categorical variables: the *p* value was calculated by the weighted chi-square test.

LC9, Life’s Crucial 9; SIRI, systemic inflammation response index; PIR, poverty income ratio; COPD, chronic obstructive pulmonary disease.

### Association between LC9, SIRI, and COPD

Table 2 demonstrates that the link between LC9 and COPD was assessed using three distinct models, all of which showed a significant negative correlation (*p* < 0.001). After controlling for variables, the prevalence of COPD decreased by 28% for every 10-point rise in LC9 in Model 3 (OR = 0.72, 95% CI: 0.67, 0.77). When divided into tertiles, the prevalence of COPD was 54% lower in the highest LC9 group (T3) than in the lowest LC9 group (T1) (OR = 0.46, 95% CI: 0.35, 0.60).

**Table 2:** Association between LC9, SIRI, and COPD, NHANES 2005–2018.

Characteristics	Model 1 [OR (95% CI)]	*p*-value	Model 2 [OR (95% CI)]	*p*-value	Model 3 [OR (95% CI)]	*p*-value
LC9-COPD						
Continuous (per 10 scores)	0.67 (0.64, 0.71)	<0.001	0.74 (0.69, 0.78)	<0.001	0.72 (0.67, 0.77)	<0.001
Tertile						
T1	1 (Ref.)		1 (Ref.)		1 (Ref.)	
T2	0.54 (0.44, 0.65)	<0.001	0.65 (0.54, 0.79)	<0.001	0.66 (0.54, 0.80)	<0.001
T3	0.30 (0.24, 0.38)	<0.001	0.46 (0.36, 0.58)	<0.001	0.46 (0.35, 0.60)	<0.001
P for trend	<0.001		<0.001		<0.001	
SIRI-COPD						
Continuous	1.31 (1.24, 1.39)	<0.001	1.18 (1.11, 1.26)	<0.001	1.17 (1.10, 1.25)	<0.001
Tertile						
T1	1 (Ref.)		1 (Ref.)		1 (Ref.)	
T2	1.49 (1.16, 1.93)	0.002	1.22 (0.95, 1.58)	0.120	1.21 (0.94, 1.56)	0.140
T3	2.03 (1.67, 2.48)	<0.001	1.45 (1.19, 1.78)	<0.001	1.41 (1.14, 1.74)	0.001
P for trend	<0.001		<0.001		0.001	

Model 1: No covariates were adjusted.

Model 2: age, sex, education level, marital status, PIR, and race were adjusted.

Model 3: age, sex, education level, marital status, PIR, race, hypertension, diabetes, and hyperlipidemia were adjusted.

LC9, Life’s Crucial 9; SIRI, systemic inflammation response index; PIR, poverty income ratio; COPD, chronic obstructive pulmonary disease; OR, odds ratio; CI, confidence interval.

**Figure 2:**
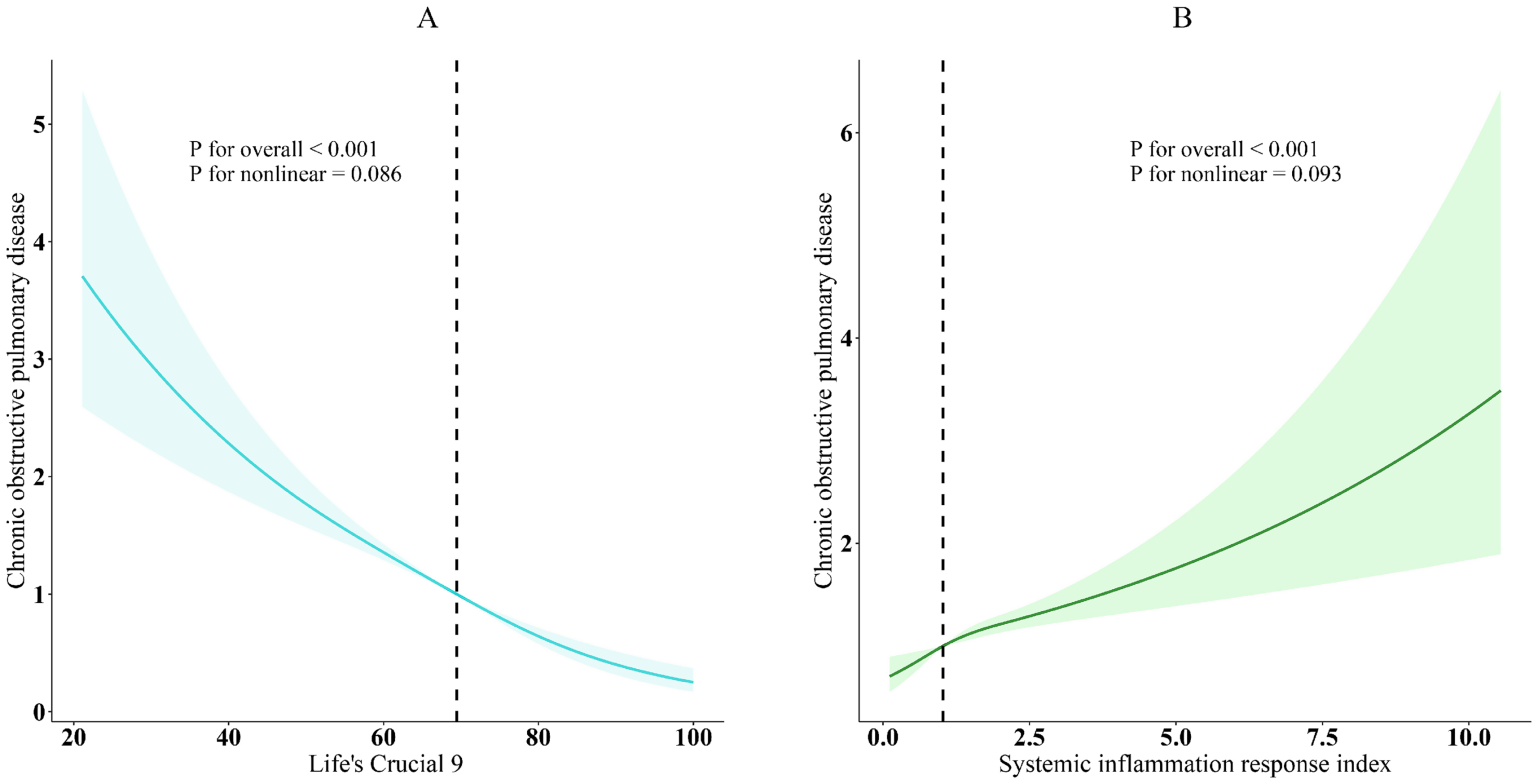
Dose–response relationships between LC9, SIRI, and COPD. **(A)** LC9-COPD; **(B)** SIRI-COPD. COPD (solid lines) and 95% confidence levels (shaded areas) were adjusted for age, sex, education level, marital status, PIR, race, hypertension, diabetes, and hyperlipidemia.

Furthermore, the association between SIRI and COPD was evaluated, and all models exhibited a strong positive association (*p* < 0.001). It showed statistically significant evidence (*p* < 0.05) that higher SIRI scores to an increased risk of COPD. After controlling variables (overall *p* < 0.001; nonlinearity *p* = 0.086), restricted cubic spline (RCS) analysis further supported a substantial linear negative correlation between LC9 and COPD prevalence, as seen in Figure 2. Given the complexity of clinical heterogeneity in patients with COPD, especially in terms of inflammation-related outcomes, we considered further adjusting for factors such as the use of anti-inflammatory drugs and the presence of other common COPD-related comorbidities (such as asthma, emphysema, chronic bronchitis, and chronic kidney disease), and the results remained robust (Supplementary Table S5).

**Figure 3:**
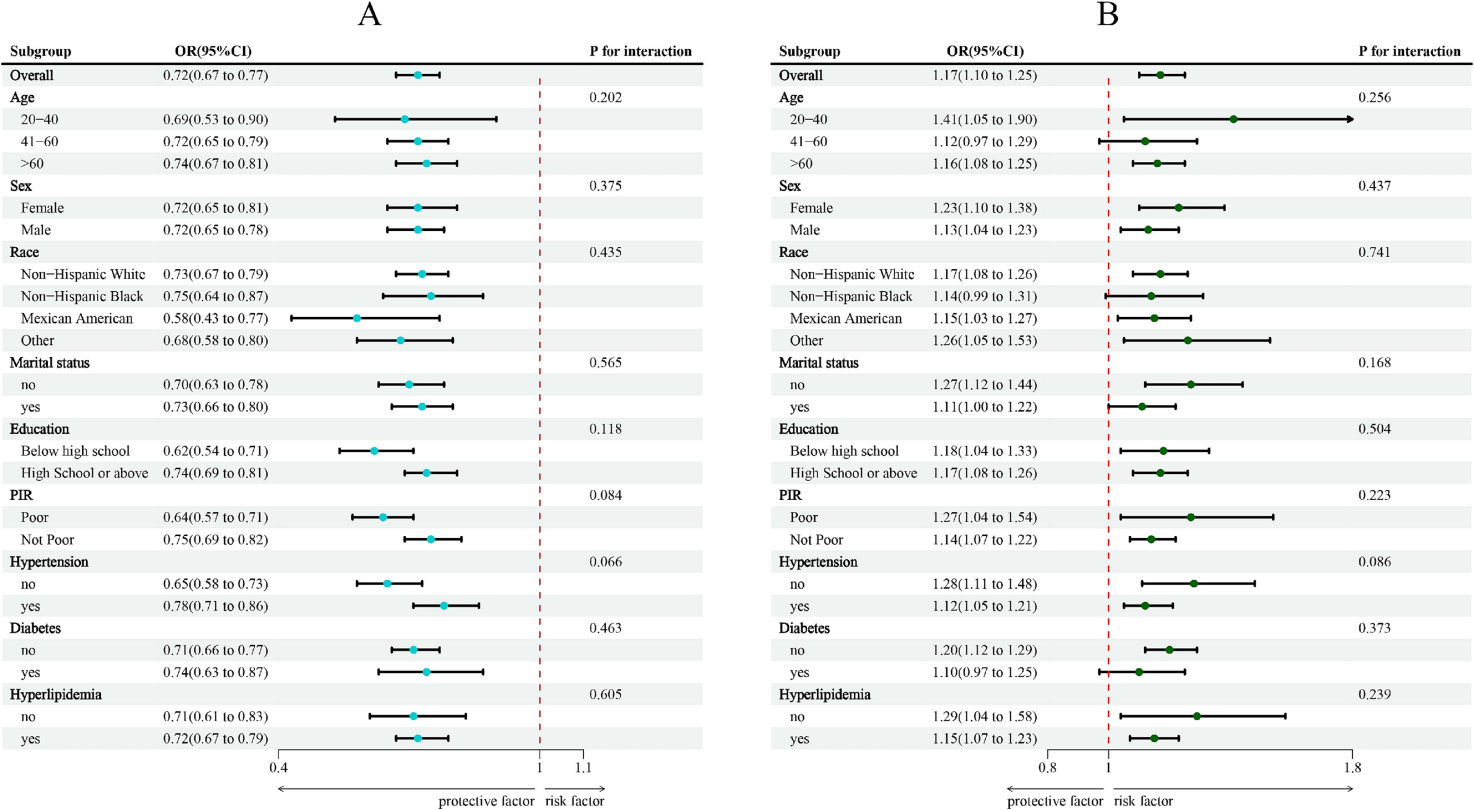
Subgroup analysis between LC9, SIRI, and COPD. **(A)** LC9-COPD; **(B)** SIRI-COPD. ORs were calculated per 10-unit increase in LC9, and each standard deviation increased in SIRI. Analyses were adjusted for age, sex, education level, marital status, PIR, race, hypertension, diabetes, and hyperlipidemia.

A strong negative correlation between LC9 and COPD was consistently found across all subgroups, according to subgroup analyses stratified by age, sex, race, marital status, education level, poverty-income ratio (PIR), hypertension, diabetes, and hyperlipidemia (Figure 3). Likewise, a significant positive association was observed between SIRI and COPD. Interaction analyses revealed no statistically significant interactions across subgroups (*p* > 0.05).

### Mediation effect

**Figure 4:**
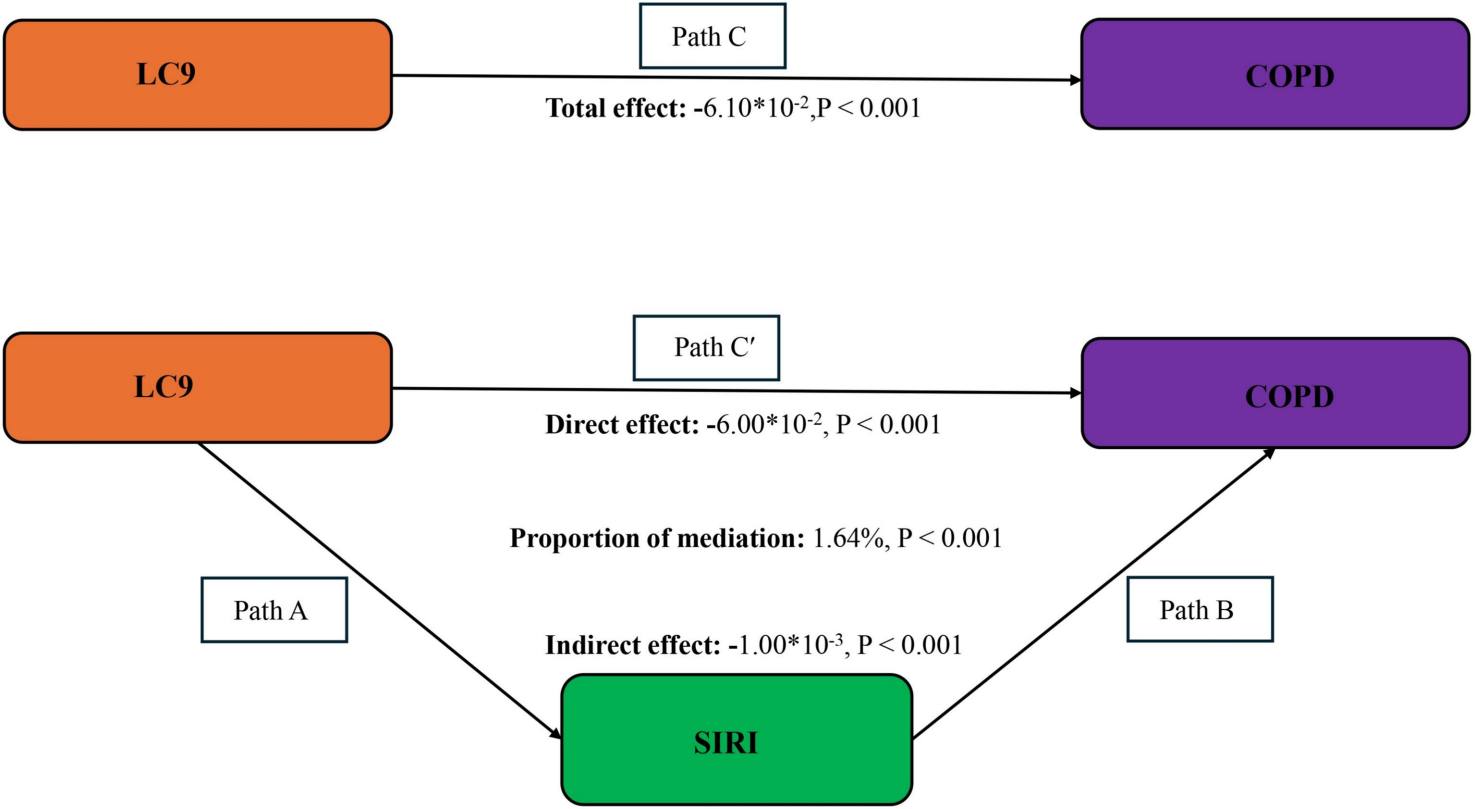
Schematic diagram of the mediation effect analysis. Path C indicates the total effect; path C′ indicates the direct effect. The indirect effect is estimated as the multiplication of paths A and B (path A*B). The mediated proportion is calculated as indirect effect/ (indirect effect + direct effect) × 100%. LC9, Life’s Crucial 9; SIRI, systemic inflammation response index; COPD, chronic obstructive pulmonary disease. Analyses were adjusted for age, sex, education level, marital status, PIR, race, hypertension, diabetes, and hyperlipidemia.

The mediation model, presented in Figure 4, explored LC9 as the independent variable, COPD as the dependent variable, and SIRI as the mediator. Following covariate adjustment, Table 3 indicates a significant relationship between LC9 and SIRI (*β* = −0.07, 95% CI: −0.08, −0.06). With a mediation proportion of 1.64% (*p* < 0.001), the mediation analysis showed a significant direct effect of LC9 on COPD (direct effect = −6.00 × 10^−2^, *p* < 0.001) and a significant indirect effect of SIRI (indirect effect = −1.00 × 10^−3^, *p* < 0.001). These results imply that SIRI plays a role in mediating the relationship between LC9 and COPD.

**Table 3:** Multivariate linear regression of LC9 and SIRI.

Characteristics	*β*	95%CI	*p*-value
LC9-SIRI	−0.07	(−0.08, −0.06)	<0.001

Adjusted for age, sex, education level, marital, PIR, race, hypertension, diabetes, and hyperlipidemia.

## Discussion

We examined data from 25,721 people in the NHANES 2005–2018 cohort for our cross-sectional analysis, including 1,257 individuals with COPD and 24,464 without COPD. Compared to COPD patients, non-COPD patients exhibited higher LC9 scores and lower SIRI levels. The results demonstrated a negative relationship between the prevalence of COPD and LC9, whereas SIRI and COPD were positively correlated. Additionally, SIRI partially mediated the association between LC9 and COPD, according to mediation analysis.

Cardiovascular diseases (CVD) are common comorbidities in COPD, and the relationship between these two conditions is complex, contributing to the increased prevalence of both and affecting symptoms and prognosis. Studies have shown that the prevalence of CVD is increased in those with COPD (15), and COPD-related mortality is linked to cardiovascular comorbidities (16). Among COPD patients, deaths due to cardiovascular diseases are more common than those caused by respiratory failure (17, 18). COPD and CVD share many common risk factors, making the control of these risk factors critical in reducing the burden of both diseases. To reduce cardiovascular disease mortality, the American Heart Association has introduced psychological health factors (including traumatic stress, chronic stress, anxiety, and depression) into the Life’s Essential 8 (LE8) scoring system, creating the new cardiovascular health score, LC9. SIRI is a new composite indicator combining the counts of neutrophils, monocytes, and lymphocytes, three distinct kinds of white blood cells, reflecting systemic inflammation, which is a hallmark of COPD, characterized by chronic airway inflammation, often involving changes in neutrophil and lymphocyte counts. For instance, in patients with AECOPD, neutrophil levels in airway cells are significantly elevated, and although neutrophil and lymphocyte counts decrease during stable periods, they remain the predominant inflammatory cells in stable COPD patients.

Higher LE8 scores lower the risk of COPD in those over 40, according to prior research that found a negative linear association between LE8 scores and COPD risk (19). SIRI has been found to be a separate risk factor for COPD (11) and is associated with exacerbations in stable COPD, predicting deterioration in COPD patients. However, the psychological health component added to LE8 has not been incorporated into these studies. Prior research indicates that LC9 is associated with various conditions, such as all-cause cardiovascular mortality (20), overactive bladder (3), infertility (4), and severe abdominal aortic calcification (21). Previous research involving 10,461 participants demonstrated that better cardiovascular health, as assessed by LC9, was associated with improved pulmonary function (Shi et al.) (5). Similarly, Guo et al. reported in a study of 14,818 participants that LC9 was linked to a reduced risk of COPD, potentially through increased lean body mass (LBM) (6). However, the inflammatory mechanisms underlying the relationship between LC9 and COPD have not yet been investigated. To address this gap, the present study is the first to evaluate the association between LC9 and COPD in a large population-based sample of 25,721 participants, and to examine the potential mediating role of SIRI. These findings may inform early preventive strategies for COPD and contribute to reducing its economic and societal burden.

The pathogenesis of COPD remains unclear, though it is often defined as having an FEV1/FVC ratio less than 0.7. The main risk factor for COPD is smoking, which induces chronic airway inflammation and oxidative stress (22). Chronic inflammation over time leads to progressive declines in lung function (23), including reductions in FVC, FEV1, and FEV1/FVC (24). Pezzuto et al. confirmed the benefits of smoking cessation in COPD patients, enhancing all respiratory function metrics, such as obstructive parameters in the large and small airways (FEV1, FEF25/75), and symptoms (mMRC score) (25).

Research has shown a close relationship between body weight and COPD. Both general obesity and abdominal obesity are associated with reduced FEV1 and FVC (26). According to a large Chinese prospective study, abdominal obesity and low body mass index (BMI) raise the risk of COPD (27). Additionally, obese individuals are often insulin resistant, leading to increased blood glucose levels. Increases in fasting blood glucose by 1 mmol/L are associated with reductions in FVC, FEV1, FVC% %, and FEV1% by 25 mL, 13 mL, 0.71–1.03%, and 0.46–0.72%, respectively (28). Compared to non-diabetic individuals, diabetic patients experience increased oxidative stress and elevated production of reactive oxygen species (ROS), and previous studies have shown that COPD development is linked to an imbalance between oxidative stress and antioxidant defenses (29). Excessive ROS production in diabetic patients can cause oxidative damage to the airways, leading to alveolar structural damage, destruction of alveolar walls, emphysema, and eventually airway airflow limitation, resulting in COPD (30). Dyslipidemia, often observed in obese and hyperglycemic patients, is also linked to inflammation, oxidative stress, and lung function (31). Elevated serum triglycerides can exacerbate respiratory resistance, while increased cholesterol levels worsen central and overall respiratory resistance (32). Dyslipidemia leads to fatty acid infiltration into macrophages, activating cytotoxic T cells and sustaining inflammatory responses (33), indirectly contributing to the development of COPD.

A healthy diet can reduce the incidence of obesity, hyperglycemia, and hyperlipidemia, potentially decreasing the prevalence of COPD. Studies have shown that antioxidant-rich diets have beneficial effects on lung function (34), with total dietary antioxidant capacity positively correlated with FEV1 and FVC (35, 36). This may help slow the decline in lung function by reducing oxidative damage. Exercise capacity is considered a key indicator of the effectiveness of pulmonary rehabilitation in COPD patients, with the six-minute walk test widely recognized as a measure of exercise capacity. Increasing evidence suggests that exercise training, including aerobic activities and resistance training, can enhance exercise endurance, muscle strength, arm function, maximal oxygen uptake, heart rate, and quality of life in COPD patients (37). Exercise may prevent the increase in macrophage and neutrophil counts in COPD mice, reducing ROS-mediated oxidative stress (38). Sufficient sleep is often associated with positive mood, while mental health disorders, especially anxiety and depression, are common comorbidities in COPD. Psychological health issues are frequently associated with high-risk lifestyle choices (e.g., smoking, high-fat, high-sugar diets), indirectly contributing to COPD development. Therefore, this study, based on the LE8 model, incorporated psychological factors and found that, compared to COPD patients, non-COPD patients had higher average mental health scores.

This research has some advantages: (1) It is the first to investigate the potential relationship between systemic inflammation and the prevalence of LC9 and COPD in the American population, suggesting that LC9 has great prospects as a preventive method for COPD. (2) SIRI, as a novel inflammation index, can predict the occurrence of COPD and provide insights for future COPD-related biomarkers. (3) The large sample size enhances the robustness of our findings, making sure that the outcomes appropriately represent the demographics of the US population.

However, this study is not without limitations. We must consider the following: (1) The only data included in our analysis is from the NHANES 2005–2018 database, which mainly shows changes among Americans. As a result, our findings might not be as applicable to different people throughout the world. (2) The existence of unmeasured variables is still a possible cause of bias even after thorough corrections for confounding variables. (3) Because this study is cross-sectional, we are unable to prove a link between the prevalence of COPD and LC9. To further understand the connection between LC9 and COPD, more prospective research is required to supply more clinical data. (4) Due to the constraints of the NHANES database, we were unable to determine whether the blood samples used to calculate SIRI were collected during stable COPD or acute exacerbation. Given that systemic inflammatory markers can vary significantly depending on the clinical phase of the disease, this limitation may affect the interpretation of the observed associations. Future studies with longitudinal data and detailed clinical information are warranted to validate our findings.

## Conclusion

In summary, we discovered a strong inverse connection between LC9 and COPD, which SIRI partially mediated. This discovery highlights the possible connection between cardiovascular health and COPD, providing fresh perspectives on COPD therapy and prevention. A comprehensive strategy that emphasizes managing inflammation and enhancing cardiovascular health may decrease the incidence of COPD.

## Data Availability

The datasets presented in this study can be found in online repositories. The names of the repository/repositories and accession number(s) can be found at: https://wwwn.cdc.gov/nchs/nhanes/.

## Footnotes

^1^ https://www.cdc.gov/nchs/nhanes/index.htm
