# Supplementary material for: Examining the mediating function of SIRI in the association between LC9 and chronic obstructive pulmonary disease
Source: Front Med (Lausanne). 2025 Jul 9;12:1606864. doi: 10.3389/fmed.2025.1606864 (PMC12283655; doi:10.3389/fmed.2025.1606864)
Supplement: Supplementary file 1 [file Table_1.docx]

**Supplementary Material**

| **Table S1.** Definition and scoring approach for the Life’s Crucial 9 score.   \| Domain \| CVH Metric \| Measurement \| Quantification and Scoring of CVH Metric \| \| --- \| --- \| --- \| --- \| \| Psychological  Health \| Depressive symptoms \| Depression Screener Questionnaire (DPQ) \| **Metric:** Nine-item depression screening instrument PHQ-9. Each symptom item in PHQ-9 is scored on a 4-point scale, from 0 (‘not at all’) to 3 (‘nearly every day’), resulting in a total score of 0 to 27 points.  **Scoring:**  Points Level  100 the score of 0 to 4 points  70 the score of 5 to 9 points  30 the score of 10 to 14 points  0 the score of 15 to 27 points \| \| Health Behaviors \| Diet \| Healthy Eating Index-2015 diet score percentile \| Quantiles of DASH-style diet adherence  **Scoring (Population):**  Points Quantile  100 ≥95^th^ percentile (top/ideal diet)  80 75^th^ – 94^th^ percentile  50 50^th^ – 74^th^ percentile  25 25^th^ – 49^th^ percentile  0 1^st^ – 24^th^ percentile (bottom/least ideal quartile) \| \| Physical activity \| Self-reported minutes of moderate or vigorous physical activity per week \| **Metric:** Minutes of moderate (or greater) intensity activity per week  **Scoring:**  Points Minutes  100 ≥150  90 120 – 149  80 90 – 119  60 60 – 89  40 30 – 59  20 1 – 29  0 0 \| \| Nicotine exposure \| Self-reported use of cigarettes or inhaled nicotine-delivery system \| **Metric:** Combustible tobacco use and inhaled NDS use; or secondhand smoke exposure  **Scoring:**  Points Status  100 Never smoker  75 Former smoker, quit ≥5 yrs  50 Former smoker, quit 1 - <5 yrs  25 Former smoker, quit <1 year, or currently using inhaled NDS  0 Current smoker  Subtract 20 points (unless the score is 0) for living with an active indoor smoker in the home \| \| Sleep health \| Self-reported average hours of sleep per night \| **Metric:** Average hours of sleep per night  **Scoring:**  Points Level  100 7 – <9  90 9 – <10  70 6 – <7  40 5 – <6 or ≥10  20 4 – <5  0 <4 \| \| Health Factors \| Body mass index \| Body weight (kg) divided by height squared (m^2^) \| **Metric:** Body mass index (kg/m^2^)  **Scoring:**  Points Level  100 <25.0  70 25.0 – 29.9  30 30.0 – 34.9  15 35.0 – 39.9  0 ≥40.0 \| \| Blood lipids \| Plasma total and HDL-cholesterol with the calculation of non-HDL-cholesterol \| **Metric:** Non-HDL-cholesterol (mg/dL)  **Scoring:**  Points Level  100 <130  60 130 – 159  40 160 – 189  20 190 – 219  0 ≥220  If the drug-treated level, subtract 20 points \| \| Blood glucose \| Fasting blood glucose or casual hemoglobin A1c \| **Metric:** Fasting blood glucose (mg/dL) or Hemoglobin A1c (%)  **Scoring:**  Points Level  100 No history of diabetes and FBG <100 (or HbA1c < 5.7)  60 No diabetes and FBG 100 – 125 (or HbA1c 5.7-6.4) (Pre-diabetes)  40 Diabetes with HbA1c <7.0  30 Diabetes with HbA1c 7.0 – 7.9  20 Diabetes with HbA1c 8.0 – 8.9  10 Diabetes with Hb A1c 9.0 – 9.9  0 Diabetes with HbA1c ≥10.0 \| \| Blood pressure \| Appropriately measured systolic and diastolic blood pressure \| **Metric:** Systolic and diastolic blood pressure (mm Hg)  **Scoring:**  Points Level  100 <120/<80 (Optimal)  75 120-129/<80 (Elevated)  50 130-139 or 80-89 (Stage I HTN)  25 140-159 or 90-99  0 ≥160 or ≥100  Subtract 20 points if treated level \|   **Table S2.** Healthy Eating Index-2015 Components & Scoring Standards.   \| Component \| Maximum points^1^ \| The standard for maximum score \| The standard for a minimum score of zero \| \| --- \| --- \| --- \| --- \| \| *Adequacy* \| \| \| \| \| Total Fruits[^2^](https://epi.grants.cancer.gov/hei/developing.html#f2) \| 5 \| ≥0.8 cup equiv. per 1,000 kcal \| No Fruit \| \| Whole Fruits[^3^](https://epi.grants.cancer.gov/hei/developing.html#f3) \| 5 \| ≥0.4 cup equiv. per 1,000 kcal \| No Whole Fruit \| \| Total Vegetables[^4^](https://epi.grants.cancer.gov/hei/developing.html#f4) \| 5 \| ≥1.1 cup equiv. per 1,000 kcal \| No Vegetables \| \| Greens and Beans[^4^](https://epi.grants.cancer.gov/hei/developing.html#f4) \| 5 \| ≥0.2 cup equiv. per 1,000 kcal \| No Dark Green Vegetables or Legumes \| \| Whole Grains \| 10 \| ≥1.5 oz equiv. per 1,000 kcal \| No Whole Grains \| \| Dairy[^5^](https://epi.grants.cancer.gov/hei/developing.html#f5) \| 10 \| ≥1.3 cup equiv. per 1,000 kcal \| No Dairy \| \| Total Protein Foods[^6^](https://epi.grants.cancer.gov/hei/developing.html#f6) \| 5 \| ≥2.5 oz equiv. per 1,000 kcal \| No Protein Foods \| \| Seafood and Plant Proteins[^6^](https://epi.grants.cancer.gov/hei/developing.html#f6)^,^[^7^](https://epi.grants.cancer.gov/hei/developing.html#f7) \| 5 \| ≥0.8 oz equiv. per 1,000 kcal \| No Seafood or Plant Proteins \| \| Fatty Acids[^8^](https://epi.grants.cancer.gov/hei/developing.html#f8) \| 10 \| (PUFAs + MUFAs)/SFAs ≥2.5 \| (PUFAs + MUFAs)/SFAs ≤1.2 \| \| *Moderation* \| \| \| \| \| Refined Grains \| 10 \| ≤1.8 oz equiv. per 1,000 kcal \| ≥4.3 oz equiv. per 1,000 kcal \| \| Sodium \| 10 \| ≤1.1 gram per 1,000 kcal \| ≥2.0 grams per 1,000 kcal \| \| Added Sugars \| 10 \| ≤6.5% of energy \| ≥26% of energy \| \| Saturated Fats \| 10 \| ≤8% of energy \| ≥16% of energy \|   (1) Intakes between the minimum and maximum standards are scored proportionately.  (2) Includes 100% fruit juice.  (3) Includes all forms except juice.  (4) Includes legumes (beans and peas).  (5) Includes all milk products, such as fluid milk, yogurt, cheese, and fortified soy beverages.  (6) Includes legumes (beans and peas).  (7) Includes seafood, nuts, seeds, soy products (other than beverages), and legumes (beans and peas).  (8) Ratio of poly- and monounsaturated fatty acids (PUFAs and MUFAs) to saturated fatty acids (SFAs).  Adequacy components represent the food groups, subgroups, and dietary elements that are encouraged. Higher scores reflect higher intakes for these components because higher intakes are desirable.  Moderation components represent the food groups and dietary elements for which there are recommended limits to consumption. For moderation components, higher scores reflect lower intakes, because lower intakes are more desirable. |
| --- | --- | --- | --- | --- | --- | --- | --- | --- | --- | --- | --- | --- | --- | --- | --- | --- | --- | --- | --- | --- | --- | --- | --- | --- | --- | --- | --- | --- | --- | --- | --- | --- | --- | --- | --- | --- | --- | --- | --- | --- | --- | --- | --- | --- | --- | --- | --- | --- | --- | --- | --- | --- | --- | --- | --- | --- | --- | --- | --- | --- | --- | --- | --- | --- | --- | --- | --- | --- | --- | --- | --- | --- | --- | --- | --- | --- | --- | --- | --- | --- | --- | --- | --- | --- | --- | --- | --- | --- | --- | --- | --- | --- | --- | --- | --- | --- | --- | --- |

**Table S3.** Definition of variables involved in this study.

| Variables | Description in NHANES |
| --- | --- |
| Age | Divided into three groups: 20-40 years old, 41-60  years old, >60 years old |
| Sex | Male and Female |
| Race | Mexican American, Non-Hispanic Black, Non-Hispanic White, Other Race |
| Educational level | Below high school, High School or above |
| Marital status | Yes: Married/Living with partner |
| PIR | Poor: <1.3; Not Poor:>=1.3 |
| Diabetes | Diabetes was defined as a history of previous diabetes, HbA1c level ≥6.5%, or fasting blood glucose level ≥126 mg/dL |
| Hypertension | The diagnostic criteria consist of self-reported hypertension history, the utilization of antihypertensive medication, a systolic blood pressure (SBP) ≥ 140mmHg, or a diastolic blood pressure (DBP) ≥ 90mmHg |
| Hyperlipidemia | (1) Triglyceride (TG) levels ≥150 mg/dl (1.7 mmol/L);(2) Total cholesterol (TC) levels ≥200 mg/dl (5.18 mmol/L);(3) Low-density lipoprotein (LDL) levels ≥130 mg/dl (3.37 mmol/L);(4) High-density lipoprotein (HDL) levels: Men: <40 mg/dl (1.04 mmol/L); Women: <50 mg/dl (1.30 mmol/L) ;(5) Individuals taking cholesterol-lowering drugs are also considered hyperlipidemia. |

PIR, Ratio of family income to poverty.

**Table S4.** Baseline characteristics of all participants were stratified by COPD, unweighted.

| **Characteristic** | **Overall**, N = 25,721 (100%) | **No**, N = 24,464 (95.1%) | **Yes**, N = 1,257 (4.9%) | **P Value** |
| --- | --- | --- | --- | --- |
| **Age (%)** |  |  |  | **<0.001** |
| *20-40* | 8,698 (35%) | 8,629 (36%) | 69 (5.4%) |  |
| *41-60* | 8,775 (38%) | 8,344 (38%) | 431 (40%) |  |
| *>60* | 8,248 (27%) | 7,491 (26%) | 757 (54%) |  |
| **Sex (%)** |  |  |  | 0.322 |
| *Female* | 13,058 (51%) | 12,509 (52%) | 549 (49%) |  |
| *Male* | 12,663 (49%) | 11,955 (48%) | 708 (51%) |  |
| **Race (%)** |  |  |  | **<0.001** |
| *Non-Hispanic White* | 11,758 (71%) | 10,923 (70%) | 835 (84%) |  |
| *Non-Hispanic Black* | 5,256 (10%) | 5,059 (10%) | 197 (6.1%) |  |
| *Other* | 4,859 (11%) | 4,699 (12%) | 160 (8.3%) |  |
| *Mexican American* | 3,848 (7.4%) | 3,783 (7.6%) | 65 (1.7%) |  |
| **Married/live with partner (%)** |  |  |  | 0.539 |
| *no* | 10,112 (36%) | 9,584 (36%) | 528 (35%) |  |
| *yes* | 15,601 (64%) | 14,872 (64%) | 729 (65%) |  |
| **Education level (%)** |  |  |  | **0.001** |
| *Below high school* | 5,684 (14%) | 5,333 (14%) | 351 (19%) |  |
| *High School or above* | 20,025 (86%) | 19,119 (86%) | 906 (81%) |  |
| **PIR (%)** |  |  |  | **0.004** |
| *Poor* | 6,949 (19%) | 6,531 (19%) | 418 (24%) |  |
| *Not Poor* | 16,817 (81%) | 16,065 (81%) | 752 (76%) |  |
| **Hypertension (%)** |  |  |  | **<0.001** |
| *no* | 14,668 (61%) | 14,204 (62%) | 464 (43%) |  |
| *yes* | 11,053 (39%) | 10,260 (38%) | 793 (57%) |  |
| **Diabetes (%)** |  |  |  | **<0.001** |
| *no* | 21,434 (87%) | 20,523 (88%) | 911 (77%) |  |
| *yes* | 4,287 (13%) | 3,941 (12%) | 346 (23%) |  |
| **Hyperlipidemia (%)** |  |  |  | **<0.001** |
| *no* | 7,180 (28%) | 6,946 (29%) | 234 (17%) |  |
| *yes* | 18,541 (72%) | 17,518 (71%) | 1,023 (83%) |  |
| **Mean LC9 score (mean (SD))** | 70.43 (13.61) | 70.81 (13.50) | 62.96 (13.76) | **<0.001** |
| **LC9, Tertile (%)** |  |  |  | **<0.001** |
| *T1* | 9,469 (32%) | 8,726 (31%) | 743 (52%) |  |
| *T2* | 8,688 (34%) | 8,338 (34%) | 350 (30%) |  |
| *T3* | 7,564 (34%) | 7,400 (35%) | 164 (17%) |  |
| **Mean psychological health score (mean (SD))** | 89.23 (23.32) | 89.50 (23.00) | 83.99 (28.42) | **<0.001** |
| **Mean HEI-2015 diet score (mean (SD))** | 39.52 (31.45) | 39.55 (31.49) | 38.94 (30.52) | 0.691 |
| **Mean physical activity score (mean (SD))** | 71.70 (41.07) | 72.22 (40.75) | 61.36 (45.60) | **<0.001** |
| **Mean tobacco exposure score (mean (SD))** | 71.33 (38.60) | 72.39 (38.27) | 50.45 (39.24) | **<0.001** |
| **Mean sleep health score (mean (SD))** | 83.45 (24.21) | 83.66 (24.00) | 79.23 (27.63) | **<0.001** |
| **Mean body mass index score (mean (SD))** | 60.54 (33.51) | 60.66 (33.50) | 58.21 (33.66) | 0.066 |
| **Mean blood lipid score (mean (SD))** | 63.91 (30.32) | 64.19 (30.33) | 58.34 (29.65) | **<0.001** |
| **Mean blood glucose score (mean (SD))** | 85.58 (24.21) | 86.06 (23.94) | 76.22 (27.55) | **<0.001** |
| **Mean blood pressure score (mean (SD))** | 68.59 (31.13) | 69.03 (31.10) | 59.93 (30.57) | **<0.001** |
| **SIRI (mean (SD))** | 1.26 (0.86) | 1.24 (0.84) | 1.55 (1.16) | **<0.001** |
| **SIRI, Tertile (%)** |  |  |  | **<0.001** |
| *T1* | 9,415 (33%) | 9,117 (34%) | 298 (23%) |  |
| *T2* | 8,206 (33%) | 7,822 (33%) | 384 (33%) |  |
| *T3* | 8,100 (33%) | 7,525 (33%) | 575 (44%) |  |

Mean (SD) for continuous variables: the P value was calculated by the weighted Students T-test.

Percentages (weighted N, %) for categorical variables: the P value was calculated by the weighted chi-square test.

Abbreviation: LC9, Life's Crucial 9; SIRI, systemic inflammation response index; PIR, poverty income ratio; COPD, chronic obstructive pulmonary disease.

**Table S5.** Association between LC9, SIRI, and COPD, NHANES 2005–2018.

| **Characteristics** | **Model 1 [OR (95% CI)]** | ***p-value*** | **Model 4**  **[OR (95% CI)]** | ***p-value*** |
| --- | --- | --- | --- | --- |
| **LC9 - COPD** |  |  |  |  |
| Continuous (per 10 scores) | 0.67(0.64,0.71) | <0.001 | 0.90(0.83, 0.98) | 0.020 |
| Tertile |  |  |  |  |
| T1 | 1 (ref.) |  | 1 (ref.) |  |
| T2 | 0.54(0.44,0.65) | <0.001 | 0.76(0.62, 0.93) | 0.010 |
| T3 | 0.30(0.24,0.38) | <0.001 | 0.51(0.38, 0.68) | <0.001 |
| *P for trend* | <0.001 |  | <0.001 |  |
| **SIRI - COPD** |  |  |  |  |
| Continuous | 1.31(1.24,1.39) | <0.001 | 1.15(1.08, 1.23) | <0.001 |
| Tertile |  |  |  |  |
| T1 | 1 (ref.) |  | 1 (ref.) |  |
| T2 | 1.49(1.16,1.93) | 0.002 | 1.19(0.92, 1.53) | 0.190 |
| T3 | 2.03(1.67,2.48) | <0.001 | 1.37(1.11, 1.69) | 0.004 |
| *P for trend* | <0.001 |  | 0.003 |  |

Model 1: No covariates were adjusted.

Model 4: age, sex, education level, marital status, PIR, race, hypertension, diabetes, hyperlipidemia, use of anti-inflammatory medications, and the presence of other common COPD-related comorbidities (Asthma, emphysema, chronic bronchitis, and chronic kidney disease) were adjusted.

Abbreviation: LC9, Life's Crucial 9; SIRI, systemic inflammation response index; PIR, poverty income ratio; COPD, chronic obstructive pulmonary disease; OR, odds ratio; CI, confidence interval.
